# A Functional Magnetic Resonance Imaging Study of Head Movements in Cervical Dystonia

**DOI:** 10.3389/fneur.2016.00201

**Published:** 2016-11-15

**Authors:** Cecília N. Prudente, Randall Stilla, Shivangi Singh, Cathrin Buetefisch, Marian Evatt, Stewart A. Factor, Alan Freeman, Xiaoping Philip Hu, Ellen J. Hess, K. Sathian, H. A. Jinnah

**Affiliations:** ^1^Department of Neurology, Emory University, Atlanta, GA, USA; ^2^Department of Rehabilitation Medicine, Emory University, Atlanta, GA, USA; ^3^Atlanta Parkinson’s Consortium Center, Atlanta VAMC, Decatur, GA, USA; ^4^Coulter Department of Biomedical Engineering, Georgia Institute of Technology, Emory University, Atlanta, GA, USA; ^5^Department of Pharmacology, Emory University, Atlanta, GA, USA; ^6^Department of Psychology, Emory University, Atlanta, USA; ^7^Rehabilitation R&D Center for Visual and Neurocognitive Rehabilitation, Atlanta VAMC, Decatur, GA, USA; ^8^Department of Human Genetics, Emory University, Atlanta, GA, USA; ^9^Department of Pediatrics, Emory University, Atlanta, GA, USA

**Keywords:** cervical dystonia, spasmodic torticollis, head movements, fMRI, isometric, cerebellum

## Abstract

Cervical dystonia (CD) is a neurological disorder characterized by abnormal movements and postures of the head. The brain regions responsible for these abnormal movements are not well understood, because most imaging techniques for assessing regional brain activity cannot be used when the head is moving. Recently, we mapped brain activation in healthy individuals using functional magnetic resonance imaging during isometric head rotation, when muscle contractions occur without actual head movements. In the current study, we used the same methods to explore the neural substrates for head movements in subjects with CD who had predominantly rotational abnormalities (torticollis). Isometric wrist extension was examined for comparison. Electromyography of neck and hand muscles ensured compliance with tasks during scanning, and any head motion was measured and corrected. Data were analyzed in three steps. First, we conducted within-group analyses to examine task-related activation patterns separately in subjects with CD and in healthy controls. Next, we directly compared task-related activation patterns between participants with CD and controls. Finally, considering that the abnormal head movements in CD occur in a consistently patterned direction for each individual, we conducted exploratory analyses that involved normalizing data according to the direction of rotational CD. The between-group comparisons failed to reveal any significant differences, but the normalization procedure in subjects with CD revealed that isometric head rotation in the direction of dystonic head rotation was associated with more activation in the ipsilateral anterior cerebellum, whereas isometric head rotation in the opposite direction was associated with more activity in sensorimotor cortex. These findings suggest that the cerebellum contributes to abnormal head rotation in CD, whereas regions in the cerebral cortex are involved in opposing the involuntary movements.

## Introduction

The dystonias are a family of neurological disorders characterized by abnormal movements and postures of different parts of the body (1, 2). Cervical dystonia (CD) is the most common, with the most frequent manifestations involving involuntary turning of the head to the right or left in the horizontal plane (rotational torticollis), often combined with tremor or jerking movements. The exact pattern of abnormal head movements is relatively stereotyped for each individual, and is chronic. The abnormal head movements are caused by excessive contraction of specific muscles of the neck, although the problem is not intrinsic to the muscles. Instead, the problem arises from abnormal neural control of neck muscles.

The brain regions responsible for abnormal head movements in CD are not entirely clear. Animal studies have revealed head movements resembling CD following manipulations of multiple regions including the basal ganglia, cerebellum, and several midbrain areas (3–7). How these findings may relate to CD in humans remain to be established. Pathological studies and physiological investigations of individuals with CD have pointed to abnormalities of the cerebral cortex, cerebellum, or vestibular pathways (8–13). Routine clinical imaging studies only rarely reveal overt anatomical defects in the brain in individuals with CD, and these are scattered across many regions such as the cerebral cortex, basal ganglia, cerebellum, midbrain, brainstem, and spinal cord (14). Quantitative voxel-based morphometric studies of CD have pointed to subtle volume changes in the basal ganglia or cerebellum (15–18). Current models suggest that CD is a network disorder involving several brain regions, although how the network is disrupted remains uncertain (19, 20).

There are no published task-based functional neuroimaging studies related to head movements in CD because these studies cannot typically be conducted when the head is moving. Instead, functional neuroimaging investigations have focused on brain abnormalities at rest, such as positron emission tomographic (PET) studies of fluorodeoxyglucose uptake (21–23) or functional magnetic resonance imaging (fMRI) of the resting state (24, 25). Other functional imaging studies of CD have used hand movements (26–30). While these studies have identified abnormalities in several brain regions even for clinically unaffected body parts, they do not address the neural control of the abnormal head movements in CD.

In a recent investigation in healthy volunteers, we demonstrated the feasibility of revealing regional changes in brain activity with fMRI during isometric head rotation (31). Isometric tasks do not involve actual movements and, therefore, provide a strategy for investigating the control of head movements *via* fMRI. The purpose of the current study was to begin to explore the neural substrates for head movements in CD. We were interested in the following questions: (1) Can direct comparisons between subjects with CD and normal controls reveal any significant group differences, considering that the baseline direction of abnormal movements in CD is heterogeneous? (2) Can the direction of abnormal movement at baseline in CD be taken into account during analyses? (3) Are there any strategies that could be used to disentangle the cause of abnormal movements from their effects in the brain? To address these questions, fMRI data were analyzed in three steps. The first step involved conducting separate within-group analyses to reveal task-related patterns of activation in subjects with CD and healthy controls. The second step comprised direct statistical comparisons between individuals with CD and controls. The final step involved a series of exploratory analyses that involved normalizing data according to the direction of rotational CD.

## Materials and Methods

### Participants

This study was carried out in accordance with the recommendations and guidelines of the Emory University Institutional Review Board with written informed consent from all subjects. All subjects gave written informed consent in accordance with the Declaration of Helsinki. Control participants were age-matched, neurologically normal, and had the ability to perform head movements in all directions. Data for the control group have already been published (31). CD participants were recruited by movement disorders neurologists at Emory University. Inclusion criteria included a diagnosis of isolated CD with a predominantly rotational abnormality, absence of any overt dystonia of the hands or other body parts, absence of tremor when lying relaxed, and absence of other significant neurological diseases. The severity of dystonia was assessed with the Toronto Western Spasmodic Torticollis Rating Scale (TWSTRS) and the Global Dystonia Rating Scale (GDRS) (32). Participants were excluded if they had significant orthopedic problems of the cervical spine, significant neck pain, contraindications for MRI, or untreated psychiatric problems. Even though involuntary head movements in CD tend to abate when lying supine with the head supported (25), participants were excluded if they had abnormal head movements when lying supine.

Because CD is a rare disorder and our focus was on a select subgroup with primarily rotational movements, it was not feasible to limit the participants to those of a single sex or with the same handedness. Instead, we attempted to balance these variables across both the CD and control groups. We did not address handedness as a covariate in the group analyses because the numbers of left-handed participants in either group (controls: *n* = 3; CD: *n* = 1) were too small for meaningful comparisons, and our previous study in healthy individuals (31) did not reveal a significant impact of handedness on results for head movements.

Participants being treated with botulinum toxin were not excluded because the vast majority of CD individuals receive this treatment, and excluding them would have yielded an atypical patient population. For those participants with CD who were being treated with botulinum toxin, scanning was conducted just before the next scheduled injection to minimize any effects of the prior treatment. On the morning of scanning, participants were instructed to delay taking any of their usual oral medications until after scans were completed.

A total of 17 individuals with CD and 18 controls were recruited. However, one control subject was excluded due to excessive head motion during all scans, and one subject with CD was excluded because of poor compliance with tasks. The final analyses therefore involved 17 controls (12 women and 5 men; 14 right-handed) and 16 individuals with CD (9 women and 7 men; 15 right-handed). Mean age was 56.8 ± 14.5 years (range 30–74 years) for the control group and 56.6 ± 11.4 years (range 31–75 years) for the CD group. All participants with CD had rotational CD. Rotational movements were rightward for 10 subjects and leftward for 6 (Table 1). Purely rotational torticollis is uncommon, so some participants also had additional involuntary movements in the coronal (laterocollis or lateral shift) or sagittal (anterocollis or retrocollis) planes.

**Table 1:** **Cervical dystonia participants**.

ID	Disease duration (years)	Direction of torticollis	Other symptoms	Overall severity of CD	Severity of torticollis	GDRS (neck)	Time since BoNT (months)
1	5	R	R-LC	18	Severe	4	NA
2	13	L	RC	20	Moderate	7	2.5
3	3	L	AC	12	Mild	4	3.4
4	15	R	L-LC	18	Slight	4	4.4
5	2	L	None	18	Moderate	5	3.2
6	13	R	None	18	Mild	4	3.3
7	5	R	R-LC	18	Slight	4	3.0
8	9	L	R-LC, R shift	17	Slight	5	3.2
9	21	R	AC	7	Mild	3	2.9
10	10	L	P shift	16	Slight	5	2.7
11	2	L	R-LC	11	Mild	3	3.3
12	13	R	AC	13	Mild	6	11.0
13	24	R	RC	15	Moderate	6	2.8
14	26	R	R-LC, AC	23	Moderate	8	4.7
15	7	R	L-LC, AC	18	Moderate	7	26.0
16	8	R	None	16	Severe	8	3.0

*The overall severity of CD was determined using the TWSTRS, while the severity of torticollis was taken only from item #1 in the same clinical scale*.

*AC, anterocollis; BoNT, botulinum toxin; GDRS, Global Dystonia Rating Scale; L, left; LC, laterocollis; NA, not applicable; P, posterior; R, right; RC, retrocollis; TWSTRS, Toronto Western Spasmodic Torticollis Rating Scale*.

### MR Scans

Functional and anatomical scans were performed at the Emory Biomedical Imaging Technology Center with a 3 T Siemens Trio TIM scanner (Siemens Medical Solutions, Malvern, PA, USA) and a quadrature transmit–receive head coil. Total scanning time was approximately 25 min. Functional images were acquired with a T2*-weighted single-shot gradient-recalled echoplanar imaging sequence with the following parameters: axial slices: 30; slice thickness: 4 mm; time to repetition (TR): 2040 ms; echo time (TE): 30 ms; flip angle: 90°; in-plane resolution: 3.4 mm × 3.4 mm; in-plane matrix: 64 × 64. Structural scans were collected after functional imaging runs with a 3D anatomic (MPRAGE) sequence (sagittal slices: 176; slice thickness: 1 mm; TR: 2300 ms; TE: 3 ms; inversion time: 1100 ms; flip angle: 8°; in-plane resolution: 1 mm × 1 mm; in-plane matrix: 256 × 256). Headphones (Etymotic Research, Elk Grove Village, IL, USA) were used for acoustic noise attenuation and to convey audio cues.

### Experimental Design

Task-based functional scans were completed during isometric head or hand tasks. All participants practiced the tasks outside the scanner prior to scanning. They were instructed to perform submaximal isometric contractions and avoid movements with the head and upper limbs. Although care was taken to select subjects with CD who could lie flat with minimal abnormal movement, all were carefully monitored during practice and scanning to ensure there were no involuntary movements of the head or other body parts.

Because our studies focused on subjects with CD who had predominantly rotational movements, we focused on isometric horizontal head rotation to the right or left. Head motion was prevented by firm foam padding around the head and restraining straps tightly placed across the forehead and chin. Eye movements were limited by asking subjects to stare at a cross projected on a screen.

Considering that hand movements have been studied extensively in healthy and CD populations, hand tasks were investigated as a positive control. Hand tasks consisted of isometric wrist extension with either hand, with the arm in a neutral position between pronation and supination. Sandbags placed outside both arms prevented actual wrist movements.

**Figure 1:**
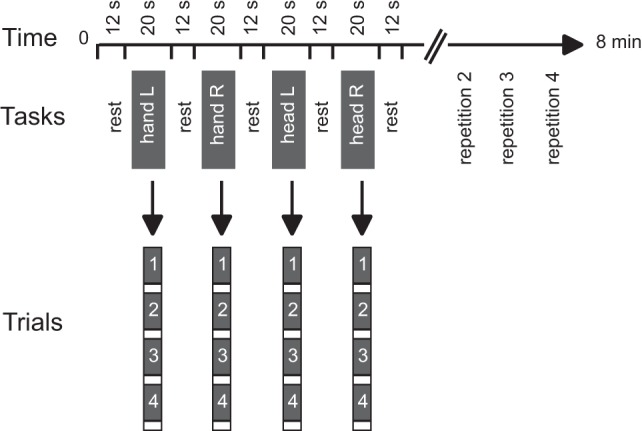
**Experimental design**. Tasks consisted of isometric head rotations to the right or left and isometric right or left wrist extensions. Each active block consisted of four trials of the same task. The sequence of active tasks’ blocks was pseudo-randomly repeated four times within each run. Modified from Prudente et al. (31).

Functional data were collected during two runs. A block design was used with alternating blocks of active tasks and rest periods (Figure 1). Each run consisted of 16 active blocks (4 per condition) interleaved with rest periods of 12.24 s in a predetermined pseudo-random sequence. Each active block included four repetitions of a single isometric task and lasted 20.4 s. The task conditions included isometric head rotation to the right, isometric head rotation to the left, isometric right wrist extension, and isometric left wrist extension. Presentation software (Neurobehavioral Systems, Albany, CA, USA) controlled the timing of audio cues for each functional run.

### Electromyography

Surface electromyography (EMG) was used to verify task performance during practice and scanning. EMG signals were recorded from the sternocleidomastoid (SCM) and extensor carpi ulnaris (ECU) muscles bilaterally during all tasks and rest periods. The SCM contributes to contralateral horizontal rotation of the head, while the ECU mediates wrist extension. Because the main goal of recording EMG was strictly to verify task performance, only a single muscle was recorded for each task. We did not plan for a full quantitative comparison between EMG and fMRI signals, because of the large number of individual muscles that would need to be evaluated for adequate assessment, and the multiple variables affecting EMG signals that could not be reliably measured such as lead location, muscle size and depth, skin impedance, and others.

MRI-compatible electrodes and Brain Vision Recorder version 1.20 (Brain Products GmbH, Munich, Germany) were used for EMG recordings at a sampling rate of 5000 Hz. Procedures and safety guidelines followed previous protocols (33). Based on the safety guidelines for recording EMG during scanning, we used the quadrature transmit–receive head coil (see MR Scans above) rather than other available coils permitting higher resolution. Brain Vision Analyser version 2.0 (Brain Products GmbH, Munich, Germany) was used for EMG signal processing, which included MR artifact correction, filtering with a low cutoff frequency of 20 Hz and signal rectification.

After data processing was completed, EMG signals were evaluated by an observer blinded to tasks and diagnosis to verify activation of the correct muscles for each task. Active and rest periods were defined based on the relative level of muscle activation in comparison to background activity. This approach was described in our previous study (31).

### Head Motion during Scans

Functional neuroimaging cannot be optimally conducted when the head is moving because head motion degrades data quality. Since our study protocol increases the risk for head movement artifacts during scans, especially in the CD group, several procedures were used to ensure that head motion during scanning was minimized. These procedures consisted of exclusion of participants with CD who could not lie still, careful instruction of participants, practicing the tasks before scans, use of firm supports to stabilize the head in the scanner, and motion correction of imaging data. To ensure that head movements were minimal, actual head motion during scanning was also quantified.

Procedures for head motion correction followed the same steps that were described in our previous study (31). In brief, the functional data were motion corrected during scanning with the built-in software Prospective Acquisition Correction or 3D-PACE (34) to compensate for translation and rotation in the *x, y*, and *z* planes. To account for any head motion that occurred between scans, intra-session alignment of functional volumes was performed in BrainVoyager QX 2.8.4 (35) after the data were collected.

We also analyzed actual head movements during scanning (31). This step involved analysis of uncorrected motion data with custom scripts in MATLAB R2014a (version 8.3.0.532, The MathWorks Inc., USA) to examine the maximal amplitude of head movements, average motion in each plane, and task-related head motion. Based on guidelines for acceptable head motion during fMRI (36), active blocks or rest periods in which head motion was greater than 1.75 mm in any plane (half the size of a functional voxel) were excluded from the final analyses, together with other task blocks and rest periods that occurred after the excessive movement. The same threshold of 1.75 mm was used for both the control and CD groups. If necessary, additional blocks within a run were also excluded to balance the number of blocks for each task and minimize potential contributions of unbalanced trial numbers to activation maps.

### Data Analysis

BrainVoyager QX 2.8.4 was used for image processing and analysis (35). Preprocessing of individual functional data included slice scan time correction with cubic spline interpolation, intra-session alignment of functional volumes with sinc interpolation, and removal of slow drifts in the data using high-pass temporal filtering to two cycles per run. Anatomic 3D images were processed, co-registered with the functional data, and transformed into Talairach space (37). For group analyses, the functional data were spatially smoothed with an isotropic Gaussian kernel (full-width half-maximum 4 mm) (38) and normalized across runs and subjects with the percent signal change transformation. Blood oxygenation level dependent (BOLD) signal time-courses were obtained by within-subject averaging of individual data points across blocks of the same type and then averaging across subjects.

Data analysis involved a three-stage, whole-brain approach to examine the patterns of activation associated with head tasks in CD. In the first stage of analysis, we performed within-group analyses to identify the regions active during isometric head or hand tasks in the control and CD groups separately. The second stage involved between-group comparisons to directly identify significant differences between subjects with CD and controls. Finally, because the dominant direction of rotation among participants with CD might be associated with significant hemispheric asymmetries, in the third stage of analysis we aimed at controlling for the side of torticollis. First, we analyzed the data for individuals with right and left torticollis separately. We also normalized their scans according to the direction of spontaneous rotation by flipping the images in the sagittal plane and reanalyzed regional brain activations. This flipping approach has been previously used by others (29, 30, 39). Within- and between-group analyses were repeated after the data were flipped to test if the dominant direction of head movement had any effects. In addition, we directly contrasted head turning in each direction to examine if moving the head in different directions was associated with distinct activation patterns.

Statistical analyses of all imaging data involved use of general linear models to assess the BOLD signal during active blocks in comparison to baseline followed by group-level analyses treating participant as a random variable. For the first (within-group) and second (between-groups) stages of data analyses, group activations during isometric head and hand tasks to either side were contrasted with the rest condition. For the third stage of analysis, isometric head tasks to either side were contrasted with head tasks to the opposite direction. All analyses used a voxel-wise significance level of *p* < 0.05, cluster-corrected for multiple comparisons with the 3D extension of the cluster-correction method (40). Results were displayed on an averaged anatomical brain for each group as in our previous study (31). MRI atlases were used for localization of activation maps with respect to 3D anatomy (41–43).

## Results

### Task Performance

Participants were able to complete the tasks adequately, as determined by observations during training and EMG signals (Table 2). For four subjects (two controls and two CDs), task performance was verified with palpation during training because EMG could not be conducted. For the remaining participants, analysis of the EMG data revealed that controls and subjects with CD were able to activate the appropriate muscles for each task on an average of 96.9 and 97.7% of all trials, respectively.

**Table 2:** **Muscle activity during scans**.

Muscle	Task	Controls Active (%)	CD Active (%)
Right ECU	Wrist extension, right	99.0	100.0
Left ECU	Wrist extension, right	97.1	94.3
Right SCM	Head rotation, left	97.1	96.6
Left SCM	Head rotation, right	94.2	100.0

*Muscle activity is shown as percent of trials in which there was obvious muscle activation in comparison to background*.

*CD, cervical dystonia; ECU, extensor carpi ulnaris; SCM, sternocleidomastoid*.

### Head Motion

**Figure 2:**
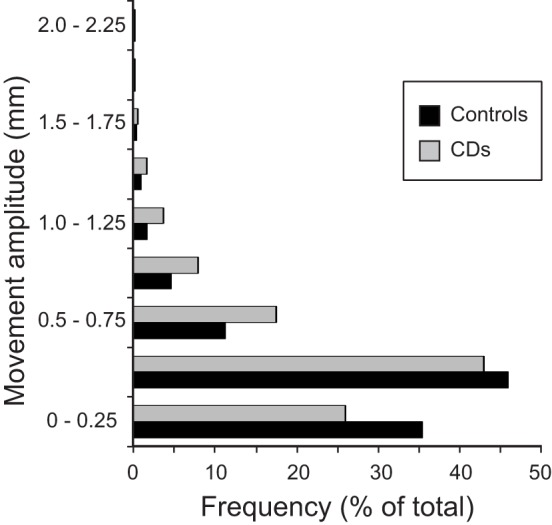
**Distribution of head movements during scanning in the control and CD groups**. The *y* axis represents the amplitude of each movement measured in mm. The *x* axis shows the distribution of head motion measurements as % of total values generated (controls: *n* = 17, total data points: 49,788; CD: *n* = 16, total data points: 41,664). Measurements for translational and rotational movements were combined. An independent samples *t*-test was performed comparing head motion (translation and rotation combined) between groups. The amplitude of head movements was significantly higher in the CD group in comparison to controls [*t*(91,450) = −48.08, *p* < 0.000, two-tailed]. Although statistically significant because of the very large number of data points analyzed, the actual magnitude of the difference was quite small (controls = 0.20 ± 0.23 mm, CD = 0.28 ± 0.27 mm), and head motion in both groups fell well below the typical threshold of 1.75 mm used for most imaging studies.

The distributions and amplitudes of uncorrected head motion were analyzed to ensure that subjects did not have excessive head movements, especially in the CD group or during isometric head tasks. For each subject, the analysis generated 1 measurement for each of the 6 movement parameters (3 planes of translation and 3 planes of rotation) associated with every brain volume collected, resulting in 103,356 data points for the whole sample. Using the threshold of 1.75 mm of head motion in any plane, we eliminated 1–3 blocks in nine subjects (three controls and six CD). This resulted in a total of 91,452 data points for the analysis of head movements. In this final sample, the vast majority of uncorrected head motion from both participants with CD and controls fell below the movement cutoff of 1.75 mm in any plane (99.9% of total data points, Figure 2). Between-group comparisons of head motion revealed that participants with CD moved more than controls (Figure 2; Table 3). However, these comparisons were overpowered (total data points: controls = 49,788; CD = 41,664), and the actual magnitude of head motion averaged 0.20 ± 0.23 mm in controls and 0.28 ± 0.27 mm in CD. Considering that head movements fell below the cutoff of 1.75 mm in any plane in both groups, artifacts in the imaging data due to head movements were likely to be minimal.

**Table 3:** **Head motion during scans**.

Movement	Task	Plane	Controls		CD	
			Mean	SD	Mean	SD
Translation (mm)	Rest	*x*	0.08	0.07	0.12	0.10
		*y*	0.11	0.12	0.16	0.15
		*z*	0.33	0.31	0.36	0.24
	Hand	*x*	0.08	0.06	0.15	0.15
		*y*	0.11	0.11	0.17	0.16
		*z*	0.32	0.30	0.46	0.32
	Head	*x*	0.10	0.08	0.13	0.10
		*y*	0.13	0.14	0.17	0.15
		*z*	0.37	0.33	0.38	0.26
Rotation (mm)	Rest	*x*	0.28	0.26	0.40	0.34
		*y*	0.16	0.17	0.19	0.17
		*z*	0.21	0.17	0.34	0.29
	Hand	*x*	0.26	0.23	0.44	0.35
		*y*	0.15	0.14	0.23	0.20
		*z*	0.21	0.16	0.42	0.37
	Head	*x*	0.31	0.31	0.39	0.31
		*y*	0.19	0.17	0.19	0.17
		*z*	0.24	0.18	0.39	0.34

*A three-way MANOVA (task × plane × group) was conducted to analyze translational and rotational head movements. There was a significant main effect for group on both translation (F = 744) and rotation (F = 2173), suggesting the CD group moved more than the control group overall. However, the actual magnitude of the difference was small and well below the 1.75 cutoff. There was a significant main effect for task on both translation (F = 140) and rotation (F = 128), suggesting one task was associated with greater motion. Post hoc analyses indicated that head tasks showed significantly more head motion than rest (p = 0.00) and hand tasks (p = 0.00). There was a significant main effect of plane on both translation (F = 8026) and rotation (F = 1644). Post hoc analyses indicated that z translation showed significantly more head motion than x (p = 0.00) and y translation (p = 0.00). For rotational movements, post hoc analyses indicated that rotation in the x plane showed significantly more head motion than the y (p = 0.00) and z (p = 0.00) planes*.

*CD, cervical dystonia*.

### First Stage of Analysis: Within-Group Analyses

**Figure 3:**
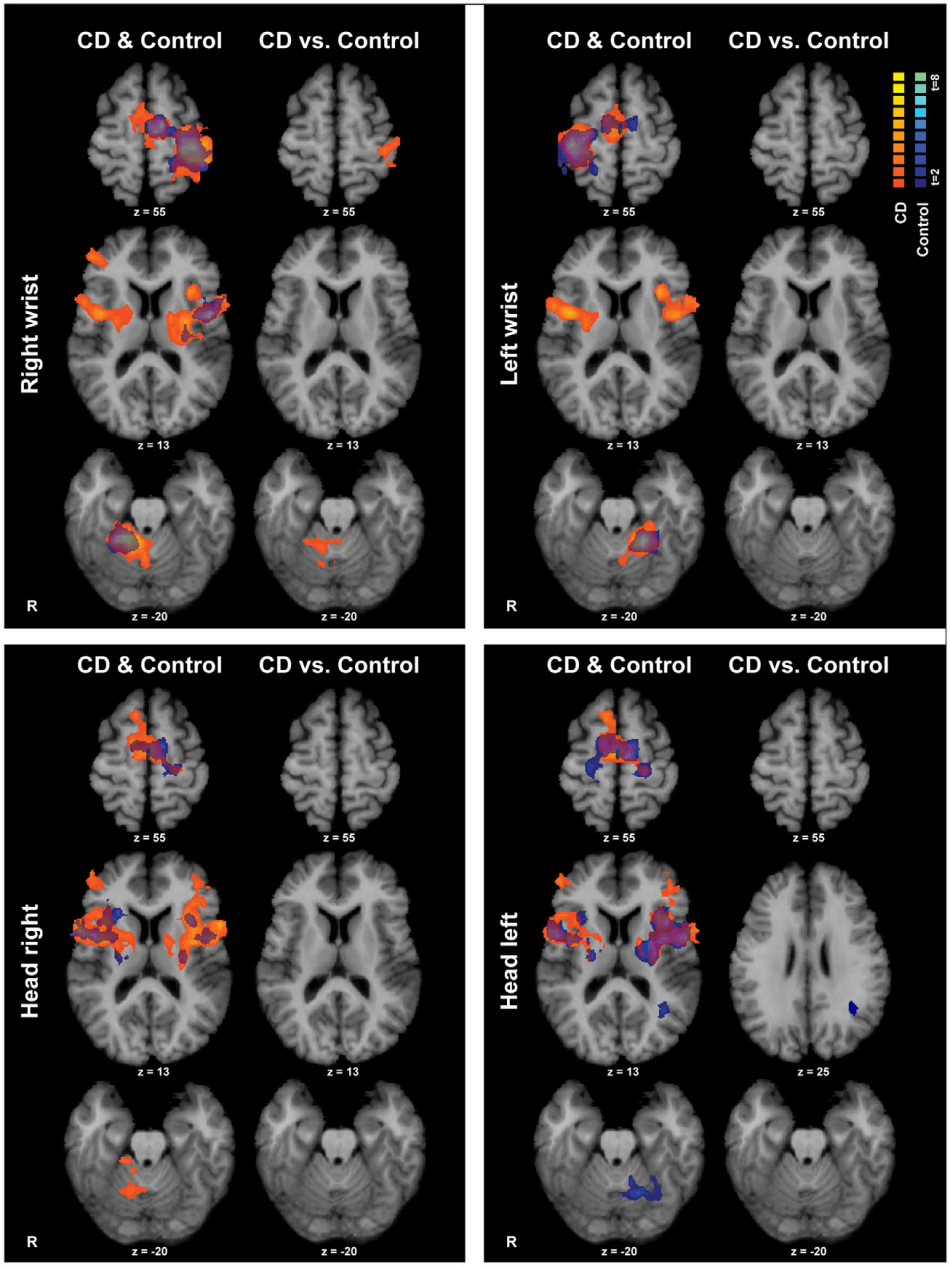
**Within- and between-groups analyses of isometric hand and head tasks**. Areas with significant activation are represented in orange for CD and in blue for controls (*p* < 0.05, random effects analysis with cluster-correction). Within-group data are shown on the left column of each panel, and between-groups results are shown on the right column. Color *t*-scales for each group are shown on the upper right corner. CD, cervical dystonia; R, right; vs., versus.

Isometric hand tasks were evaluated first to provide a positive control and activation landmarks for subsequent comparison of head tasks. Details of fMRI activations for isometric hand and head tasks for healthy individuals are presented elsewhere (31) and are summarized in blue in Figure 3.

Isometric wrist extension with either hand in comparison to baseline among participants with CD activated similar brain regions as in controls, including the contralateral precentral gyrus in the area known as the hand knob (44), the contralateral postcentral gyrus, bilateral supplementary motor area (SMA), bilateral basal ganglia, and ipsilateral cerebellum (Figure 3; Table 4). These results imply that the isometric hand tasks were an appropriate control condition in both groups. Given that our goal was to investigate the neural substrates for head movements in CD, the remainder of the manuscript focuses on isometric head tasks.

**Table 4:** **Isometric head tasks vs. baseline in CD**.

Isometric task	Region	Hemi	*x*	*y*	*z*	*t*_max_
Head rotation, right	Medial precentral gyrus	L	−21	−25	55	3.07
	Lateral/ventral precentral gyrus	L	−51	2	22	3.79
	Lateral/ventral precentral gyrus	R	54	1	−37	4.35
	SMA	L	−3	−10	58	4.54
	SMA	R	3	−13	55	4.11
	Pre-SMA	L	−3	−7	52	4.45
	Pre-SMA	R	6	8	49	3.66
	Anterior cingulate gyrus	R	4	14	31	3.08
	Middle cingulate gyrus	L	−9	−1	40	3.16
	Middle cingulate gyrus	R	13	11	37	3.26
	Putamen	L	−27	−13	10	4.45
	Putamen	R	24	−7	10	4.42
	Globus pallidus	L	−15	2	4	3.46
	Globus pallidus	R	15	−1	7	4.03
	Ventrolateral thalamus	L	−15	−13	7	3.04
	Middle frontal gyrus	L	−42	38	25	3.84
	Middle frontal gyrus	R	39	44	19	4.21
	Anterior insula	L	−30	14	13	5.25
	Anterior insula	R	36	14	16	4.77
	Mid-insula	L	−33	2	10	5.99
	Mid-insula	R	39	−1	10	4.82
	Frontal operculum	L	−45	11	4	7.26
	Frontal operculum	R	54	5	16	6.74
	Parietal operculum	L	−45	−37	31	3.47
	Parietal operculum	R	54	−31	22	3.75
	Postcentral gyrus	L	−57	−25	22	4.17
	Postcentral gyrus	R	54	−19	22	3.91
	Cerebellum, lobule III	R	21	−34	−20	3.26
	Vermis, lobule V	R	0	−58	−17	3.09
	Cerebellum, lobules V−VI	R	15	−55	−17	3.21
	Dentate nucleus	R	12	−34	−29	5.65
Head rotation, left	Medial precentral gyrus	L	−21	−25	55	3.47
	SMA	R	3	−13	55	4.93
	SMA	L	−6	−13	61	4.93
	Middle cingulate gyrus	L	−9	−4	40	2.81
	Putamen	R	24	−10	16	4.69
	Putamen	L	−30	−10	7	4.01
	Globus pallidus	R	18	−4	4	3.07
	Globus pallidus	L	−21	−7	7	3.79
	Ventrolateral thalamus	R	12	−10	4	2.61
	Ventrolateral thalamus	L	−15	−7	10	4.99
	Middle frontal gyrus	R	36	41	22	4.98
	Middle frontal gyrus	L	−36	26	31	4.90
	Anterior insula	R	42	17	4	4.40
	Anterior insula	L	−30	14	13	5.00
	Mid-insula	R	36	−1	13	4.65
	Mid-insula	L	−39	2	7	6.54
	Frontal operculum	R	45	17	10	5.11
	Frontal operculum	L	−51	−1	13	5.67
	Parietal operculum	R	51	−28	34	4.47
	Postcentral gyrus	R	54	−22	28	4.12

*Group activations were analyzed using a voxel-wise significance level of *p* < 0.05, corrected for multiple comparisons with a 3D extension of the cluster-correction method. Significant activation foci are shown in Talairach coordinates (37). Cluster sizes for each contrast: head right > rest = 102; head left > rest = 89. Contrasts that did not reach statistical significance are not shown*.

*Hemi, hemisphere; L, left; R, right; SMA, supplementary motor area*.

Isometric head rotation to the right or left in comparison to rest also produced overall patterns of activation in subjects with CD that were similar to those in controls (Figure 3; Table 4). Both groups showed activation of the precentral gyrus, SMA, basal ganglia, anterior/mid-insula, frontal and parietal operculum, and ipsilateral cerebellum.

### Second Stage of Analysis: Between-Group Comparisons

The CD and control groups were then compared directly to determine any differences between groups during the head tasks (Figure 3). Comparisons for isometric head rotation to the right revealed no statistically significant differences between groups. For isometric head rotation to the left, there was greater activity in controls in comparison to participants with CD only in a small region of the ipsilateral angular gyrus.

The lack of more robust differences between the participants with CD and controls could reflect two possibilities. One possibility is that the magnitude of any differences between the groups was too small to reach the stringent statistical thresholds used for data analysis. Another possibility is raised by the fact that involuntary head movements in CD occur in a consistently patterned direction for each individual (45). This directional preference may be associated with significant asymmetry of brain activity. If this is the case, then combining individuals with right or left torticollis in the same group analysis might have obscured asymmetrical abnormalities in the CD group.

### Third Stage of Analysis: Directional Preference of Horizontal Rotation among CD Subjects

To address the hypothesis that the direction of involuntary head movements in CD might produce significant asymmetries, we reanalyzed the CD data taking this direction, referred to here as the “pathological” direction, into consideration. We hypothesized that any asymmetries between head movements in the pathological and non-pathological directions might reveal which regions contribute to the involuntary turning movements and which regions might be involved in opposing the involuntary movements. In principle, voluntarily turning toward the pathological direction would reveal the combined effect of both voluntary and involuntary movements. In contrast, voluntarily turning away from the pathological direction might require more voluntary effort to oppose the involuntary tendencies.

First, we divided the CD group into those with right (*n* = 10) or left (*n* = 6) torticollis and contrasted each subgroup separately with matched numbers of control subjects. These analyses failed to yield any robust differences (data not shown). The absence of clear abnormalities may be either because no such abnormalities exist or because the numbers of cases for either direction were too small to produce a statistically meaningful result.

To attempt to resolve these possibilities, we used a second strategy to address the potential consequences of the pathological head direction in CD that involved digitally reversing the structural and functional datasets of participants with CD who had left torticollis to match those with right torticollis (29, 30, 39). Data normalized in this manner are referred to hereafter as “CD_PATH_.”

The within-group analysis for the CD_PATH_ data did not considerably change the previously observed activation maps when comparing hand tasks to rest (not shown), suggesting that flipping the imaging data and tasks for participants with CD who had left torticollis did not result in profound distortion of the overall findings, with only small changes that were likely due to statistical threshold effects.

For the within-group analysis of the CD_PATH_ data for isometric head rotation in the direction of pathological movement in comparison to rest, there was bilateral cerebellar activation (instead of ipsilateral in the non-normalized data; not shown). No notable differences were observed for isometric head rotation in the non-pathological direction in comparison to rest (compared to the non-normalized data; not shown). These results suggest more prominent activation of the cerebellum when individuals with CD attempt to move the head in the pathological direction.

Direct statistical comparisons between controls and the CD_PATH_ groups were also conducted to determine whether normalizing the CD data according to the direction of involuntary head movements would reveal differences between groups. For isometric head rotation to the right (pathological direction for CD_PATH_) in comparison to baseline, the CD_PATH_ group showed greater activity in comparison to controls ipsilaterally in the SMA, and contralaterally in the postcentral gyrus and inferior frontal gyrus (data not shown). For isometric head rotation to the left (non-pathological direction for CD_PATH_) in comparison to rest, there were no statistically significant differences between CD_PATH_ and control participants (data not shown). These findings suggest that there were differences between controls and CD for head movements in the pathological direction, whereas no differences between groups were detected when comparing head movements in the non-pathological direction.

**Figure 4:**
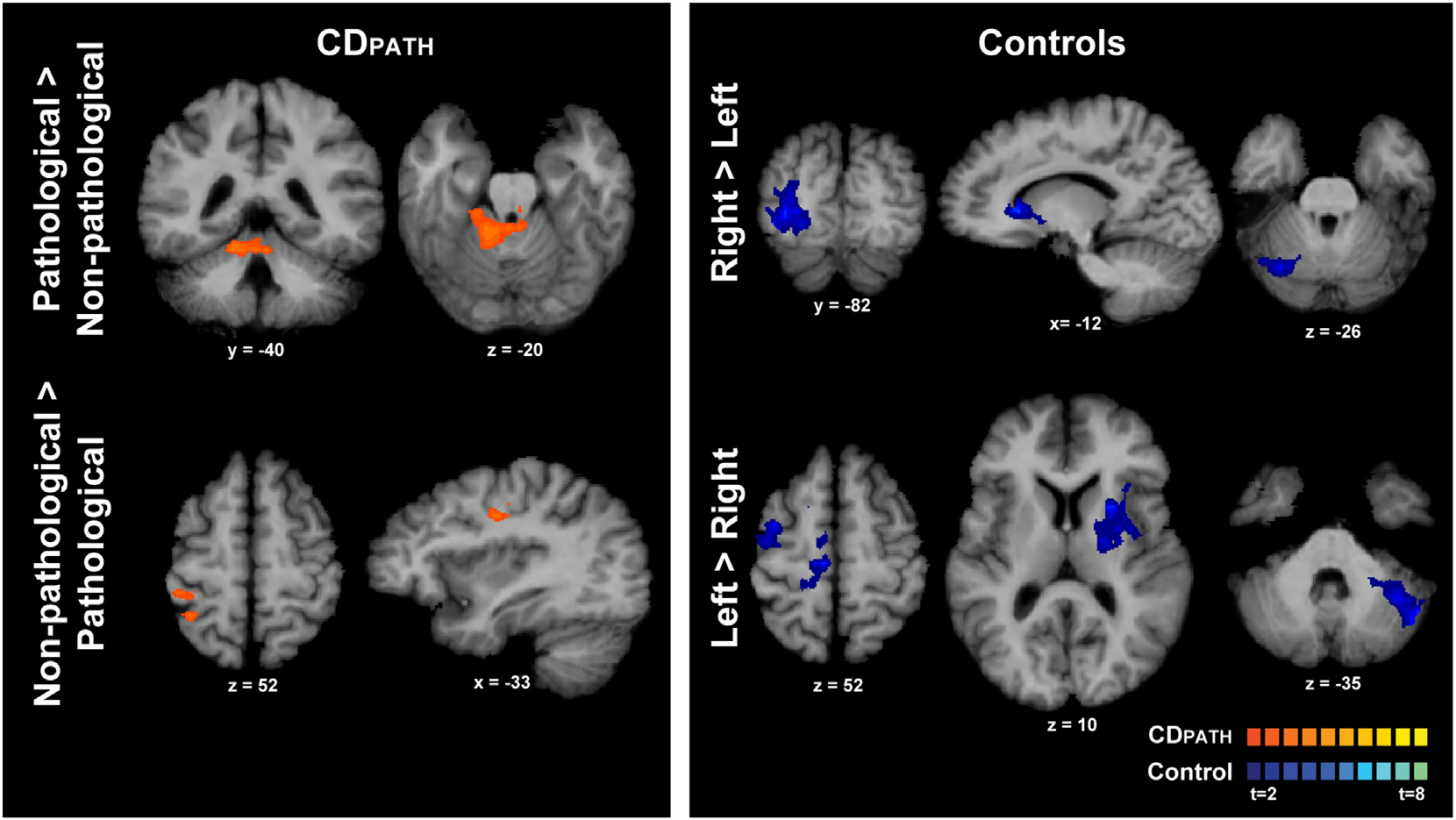
**Within-group comparisons of isometric head rotation in different directions in CD_PATH_ and controls**. Areas with significant activation are represented in orange for CD_PATH_ and in blue for controls (*p* < 0.05, random effects analysis with cluster-correction). The images on the top row show regions with greater activity for the direction of torticollis in comparison to the non-pathological direction (or head rotation right in comparison to left in controls). The images on the bottom row show results for regions with greater activity for non-pathological direction of head movement in comparison to the pathological direction (or head rotation left in comparison to right in controls). Color *t*-scales for each group are shown on the lower right corner. CD_PATH_, cervical dystonia group normalized to the direction of pathological movement; R, right.

We then conducted direct contrasts within the CD_PATH_ group for isometric head rotation in the pathological vs. non-pathological directions. We hypothesized that any significant brain asymmetries would be exaggerated on this contrast. Isometric head rotation in the pathological direction contrasted with that in the non-pathological direction produced significant activation only in the ipsilateral anterior cerebellum and vermis, in lobules III–V (Figure 4; Table 5). Conversely, isometric head rotation in the non-pathological direction compared with head rotation in the pathological direction was associated with activation in a few cerebral cortical regions, including cortex near the junction of the superior frontal and precentral sulci (where the frontal eye field is located) contralateral to the pathological direction, and, in the opposite hemisphere, the postcentral sulcus and supramarginal gyrus (46) (Figure 4; Table 5). These results imply significant asymmetries of brain activity associated with the pathological and non-pathological directions in CD.

**Table 5:** **Within-group comparisons for isometric head rotation to different directions in CD_PATH_ and controls**.

Group	Isometric task	Region	Hemi	*x*	*y*	*z*	*t*_max_
CD_PATH_	Pathological > non-pathological	Vermis, lobule III–IV	R	3	−40	−20	3.63
		Cerebellum, lobules III−V	R	15	−40	−20	4.35
	Non-pathological > pathological	Postcentral gyrus	R	30	−46	52	3.67
		Precentral sulcus	L	−33	−10	34	3.80
Control	Head right > head left	Superior/middle occipital cortex	R	30	−82	−5	4.31
		Caudate nucleus	L	−12	20	4	4.87
		Cerebellum, lobule VI−crus I	R	30	−64	−26	3.81
	Head left > head right	SMA	R	6	−13	64	4.90
		Premotor area	R	33	−13	49	4.36
		Precentral gyrus	R	21	−25	61	3.07
		Posterior cingulate gyrus	R	6	−34	31	4.62
		Putamen/thalamus/insula	L	−21	8	10	4.31
		Cerebellum, lobule crus I−II	L	−33	−52	−35	3.80

*Group activations were analyzed using a voxel-wise significance level of *p* < 0.05, corrected for multiple comparisons with a 3D extension of the cluster-correction method. Significant activation foci are shown in Talairach coordinates (37). Cluster sizes for each contrast: pathological > non-pathological = 39; non-pathological > pathological = 39; head right > head left = 52; head left > head right = 54. Contrasts that did not reach statistical significance are not shown. CD_PATH_, cervical dystonia normalized to side of torticollis*.

*Hemi, hemisphere; L, left; R, right; SMA, supplementary motor area*.

To confirm these findings, we reversed the strategy for the CD_PATH_ group. Specifically, data from the right torticollis group were digitally reversed to match the left torticollis group. Comparisons for head rotation in the pathological vs. non-pathological directions provided a mirror image of the complementary flip (data not shown), raising confidence the CD_PATH_ results are reproducible.

Finally, we contrasted activations for rightward vs. leftward head rotation in the control group to provide some context for the above contrast of head rotation in the pathological vs. non-pathological direction in participants with CD (note that the latter contrast in CD equates to a contrast of rightward vs. leftward head rotation following normalization). In controls, the contrast of rightward vs. leftward head rotation revealed greater activity in the right superior/middle occipital cortex, left caudate nucleus, and right posterolateral cerebellum (Figure 4; Table 5). The opposite contrast, leftward vs. rightward head rotation, yielded greater activity in right precentral gyrus, premotor, SMA and cingulate cortex, and a contiguous belt of activation extending from left anterior insular cortex into the left putamen and thalamus, as well as in the left lateral cerebellum (Figure 4 and Table 5). These findings indicated that different patterns of activation emerged when controls moved the head in opposite directions and, furthermore, these patterns did not resemble the activation maps observed in the CD_PATH_ data.

## Discussion

This study used a novel approach with isometric tasks to explore regional brain activity in participants with CD. Our study is one of the largest task-fMRI studies of CD to be conducted, with the most uniform population of subjects, i.e., with mostly rotational head abnormalities. Compliance with tasks was verified by EMG during fMRI scanning. Head motion was measured in all three planes, found to be within accepted thresholds for neuroimaging studies, and corrected for final analyses. Overall, participants with CD showed similar patterns of brain activation as controls for both isometric hand and head tasks, and only a few of the regional differences reached statistical significance in between-group comparisons. However, when results from individuals with CD were normalized according to the direction of pathological head rotation, significantly asymmetrical brain activation patterns emerged. Isometric head rotation in the pathological direction was associated with more prominent activation of the anterior cerebellum, whereas isometric rotation in the opposite direction was associated with activation of sensorimotor areas of the cerebral cortex. Although the lack of differences between groups was unexpected, the fact that distinct brain patterns were observed after normalizing the CD data according to the direction of pathological head rotation has important implications for future studies addressing the pathogenesis of abnormal movements in CD and other focal dystonias. Furthermore, the findings provide an important framework for future hypothesis-driven investigations focused on the neural substrates of head movements in CD.

### Distinguishing Cause from Effect

One of the limitations inherent to neuroimaging studies is that it is challenging to distinguish the brain region that may cause an abnormality from secondary effects (47). These secondary effects may reflect relatively short-term reactive changes in brain activity, such as the nearly instantaneous alterations in sensory feedback following an abnormal movement. Alternatively, they may reflect long-term adaptations to a chronically abnormal process. Both of these influences are relevant when considering CD, which is likely to be associated with short-term as well as long-term changes in sensory feedback and patterns of brain activity.

We attempted to discriminate causal from secondary effects by exploring the functional imaging data in relation to the direction of spontaneous abnormal rotations in the CD group. Because the involuntary movements in CD are chronically patterned in a single predominant direction for each patient, we hypothesized that attempts to voluntarily turn the head might reveal hemispheric asymmetries associated with the abnormal involuntary movement. More specifically, voluntarily turning the head in the same direction as the pathological direction of torticollis may require less volitional effort because of the inherent tendency of the involuntary mechanisms. Thus, comparing activation patterns for isometric movements toward vs. away from the pathological direction might point more specifically to the regions responsible for the abnormal movements. Interestingly, this analysis revealed prominent activity of the ipsilateral anterior cerebellum (Figure 4), which plays a key role in motor control (48) and has been implicated as playing a causal role in dystonia (19, 47). If this interpretation is correct, our results imply that the cerebellum may be the primary driver of abnormal head rotation in CD.

By contrast, turning the head opposite to the pathological direction would presumably require more volitional effort to antagonize the inherent tendency of the involuntary mechanisms in CD. It may also be associated with more prominent sensory feedback from proprioceptors in muscles that fail to relax. Consistent with this idea, this analysis revealed significant activation in the frontal eye field contralateral to the pathological direction, and, in the opposite hemisphere, the ipsilateral postcentral sulcus, and supramarginal gyrus (Figure 4). Although proprioceptive information is processed in all subfields of primary somatosensory cortex, Brodmann’s area 3a (in the depth of the central sulcus) seems to be most important, followed by Brodmann’s area 1 and 2 (the latter located in the postcentral sulcus) (49). The middle cingulate gyrus is activated primarily in relation to movement execution, while the regions surrounding the frontal eye fields are premotor areas involved in motor planning (46). Thus, our results suggest that moving the head opposite to the pathological direction involved increased processing in sensorimotor regions, which may reflect a compensatory adaptation to the involuntary rotational movements.

These interpretations are consistent with several prior studies in CD. Clinical imaging studies using computed tomography and structural MRI have linked CD with focal lesions of cerebellar circuits (14, 47, 50, 51). A diffusion tensor imaging (DTI) study in individuals with craniocervical dystonia (dystonia of facial and neck muscles combined) indicated abnormal cerebellar microstructure and fiber organization, especially in the anterior cerebellum and vermis (52). Another DTI study showed that persons with CD had decreased axonal fiber organization in the superior cerebellar peduncles, which carry the output fibers from the cerebellum to the thalamus and brainstem (53). Voxel-based morphometry studies in CD have demonstrated abnormalities in cerebellar gray matter volume, including the anterior cerebellum (15, 17, 52, 54, 55). PET studies have indicated increased glucose metabolism in the cerebellar hemispheres bilaterally (22). Collectively, these imaging investigations suggest abnormal structure and function of the cerebellum and its connections in CD. However, the cerebellum is not the only region implicated, as several studies have suggested involvement of other regions such as the basal ganglia and sensorimotor cortex (54).

### Study Limitations

Although our results provide novel insights into the patterns of brain activity associated with head movements in CD, some limitations must be noted. First, neuroimaging studies rarely allow for conclusive discrimination of cause from effect, as noted above. A second, related problem is that the abnormal head movements in CD are largely involuntary, while intentional isometric contractions are voluntary. Thus the results presented here are likely to reflect an interaction between voluntary and involuntary brain mechanisms.

Another limitation is that fMRI is not ideal for detecting changes in relatively small brain regions that have been proposed to play a role in the abnormal head movements of CD, such as the interstitial nucleus of Cajal (4), superior colliculus (6), or red nucleus (3). On a related note, our imaging window extended from the cerebral cortex through most of the cerebellum, but the most caudal regions of the cerebellum and brainstem fell outside of the imaging window. Therefore, no comments can be made regarding those regions.

A third limitation is that even though both groups showed visually different activation patterns for isometric head rotation to either side (un-flipped data) in the within-group analyses, statistical comparisons between groups failed to show significant differences. The most likely explanation for this discrepancy is that any differences between CD and controls were small in the case of the isometric tasks studied here, so that they did not survive correction for multiple comparisons. In support of this possibility, we did observe group differences using uncorrected maps. The ideal study would require a much larger group of participants with CD, all with pure rotational torticollis to one side, as well as a replication study using a similar but independent cohort. However, recruiting two large cohorts with CD is challenging because CD is so rare, and such a study is even more difficult when employing strict inclusion and exclusion criteria to control for handedness and direction of head rotation.

A final limitation is that quantitative EMG data could not be analyzed in relation to imaging results, because of the limited numbers of muscles studied. Because abnormal head movements are a cardinal feature of CD, it is very likely that EMG would reveal group differences between CD and healthy controls. How these differences relate to voluntary vs. involuntary activity is not clear, so EMG may not be an appropriate surrogate measure for voluntary effort in the CD population. However, our main goal in using EMG was not to quantitatively characterize muscle activation patterns, but to use it to verify task compliance. In this regard, the additional acquisition of EMG data was very useful.

### A Novel Model for Pathogenesis of Abnormal Movements in CD

Although it is challenging to conclusively distinguish cause and effect from imaging data, the abnormal activation maps seen in CD can be accommodated by a novel conceptual model. This model must be considered speculative, and one that will require additional studies to test more critically. In this model, abnormal asymmetric drive originating from the cerebellum itself or its connections is responsible for the frequent involuntary rotation of the head to one side or the other. These abnormal movements are then partly counteracted by forebrain mechanisms that attempt to correct the abnormal head posture. Thus, the dynamic position of the head observed in CD may reflect an interaction between two mechanisms involved in motor control; a pathological one and a compensatory one.

This proposed mechanism is analogous to the model accepted to underlie gaze-evoked eye nystagmus, where abnormal slow drifting movements of the eyes from a target (usually caused by cerebellar dysfunction or brainstem dysfunction) are constantly being corrected by rapid saccades that bring the eyes back on target (mediated by forebrain structures involving cortex and basal ganglia) (20, 45). This model, in which abnormal head movements in CD reflect abnormal interactions between multiple brain regions involved in motor control, fits with the idea that dystonia is a network disorder caused by abnormal interactions between brain regions (19, 47) and may help explain why different studies sometimes place the primary region of brain abnormality in different areas.

## Conclusion

We used a novel isometric head task during functional neuroimaging to explore the neural substrates of head movements in CD. In one of the largest and most uniform cohorts of CD with predominantly rotational torticollis studied with task-fMRI to date, we found no significant differences except when the data were evaluated in relation to the direction of abnormal dystonic rotation. Although it is customary in imaging studies to lump all subjects with CD together to obtain sufficient numbers of cases for a meaningful result (because the disorder is rare), the strategy used here implies that a more refined subtyping may be helpful for revealing underlying abnormalities. The results suggest that the pathological head movements in CD are associated with abnormal activation of the cerebellum, while other forebrain regions may be involved in compensations. A replication study focusing specifically on the novel method used here, and perhaps controlling for other variables such as direction of head rotation at rest and handedness would be valuable. Nonetheless, the current findings are relevant for understanding the neuroanatomical substrates for abnormal head movements in CD, and for future studies addressing heterogeneity among the dystonias.

## Author Contributions

CP designed research, performed experiments, analyzed data, and wrote the manuscript. RS performed experiments, analyzed data, and reviewed the manuscript. SS analyzed data and reviewed the manuscript. CB designed research and reviewed the manuscript. ME, SAF, and AF performed experiments and reviewed the manuscript. XH designed research and reviewed the manuscript. EH designed research, analyzed data, and reviewed the manuscript. KS and HJ designed research, analyzed data, and wrote the manuscript.

## Conflict of Interest Statement

The authors declare that the research was conducted in the absence of any commercial or financial relationships that could be construed as a potential conflict of interest. The reviewer (GM) and handling Editor declared their shared affiliation, and the handling Editor states that the process nevertheless met the standards of a fair and objective review.

## Abbreviations

AC, anterocollis; BOLD, blood oxygenation level dependent; BoNT, botulinum toxin; CD, cervical dystonia; CD_PATH_, cervical dystonia normalized to side of torticollis; ECU, extensor carpi ulnaris; EMG, electromyography; fMRI, functional magnetic resonance imaging; GDRS, Global Dystonia Rating Scale; Hemi, hemisphere; L, left; LC, laterocollis; MTG, middle temporal gyrus; NA, not applicable; P, posterior; PET, positron emission tomography; R, right; RC, retrocollis; SCM, sternocleidomastoid; SMA, supplementary motor area; TE, echo time; TR, time to repetition; TWSTRS, Toronto Western Spasmodic Torticollis Rating Scale.
